# Role of risk and protective factors in risky sexual behavior among high school students in Cambodia

**DOI:** 10.1186/1471-2458-10-477

**Published:** 2010-08-12

**Authors:** Siyan Yi, Krishna C Poudel, Junko Yasuoka, Paula H Palmer, Songky Yi, Masamine Jimba

**Affiliations:** 1 Department of Community and Global Health, Graduate School of Medicine, the University of Tokyo, Japan, 7-3-1 Hongo, Bunkyo-ku, Tokyo, Japan; 2 School of Community and Global Health, Claremont Graduate University, Claremont, California, USA, 18 East Via Verde, Ste.100, Claremont, CA 91773, USA; 3 Battambang Provincial Department for Education, Youth, and Sports, Ministry of Education, Youth, and Sports, Cambodia, Road No 57, Phoum Damnak Luong, Sangkat Wat Kor, Battambang Provincial Town, Cambodia

## Abstract

**Background:**

In many developing countries, adolescents have become increasingly prone to engage in habitual risky sexual behavior such as early sexual initiation and unprotected sex. The objective of this study was to identify the operation of risk and protective factors in individual, family, peer, school, and community domains in predicting risky sexual behavior among male and female adolescents in Cambodia.

**Methods:**

From October 2007 to January 2008, we collected data from 1,049 students aged 14 to 20 years. Risky sexual behavior was measured using a scale consisting of four items: sexual intercourse during the past three months, number of sex partners during the past three months, age at first experience of sexual intercourse, and use of condom in last sexual intercourse. The risk factors examined included substance use, depression, peer delinquency, family violence, and community violence. Studied protective factors included family support function, frequency of family dinner, and school attachment.

**Results:**

Of the 1,049 students surveyed, 12.7% reported sexual intercourse during the past three months. Out of those sexually active students, 34.6% reported having two or more sex partners over the same period, and 52.6% did not use a condom during their last sexual intercourse. After controlling for other covariates, a higher likelihood of risky sexual behavior remained significantly associated among male participants with higher levels of substance use, higher levels of peer delinquency, and higher family income. In contrast, risky sexual behavior did not retain its associations with any of the measured protective factors among male participants. Among female participants, a higher likelihood of risky sexual behavior remained significantly associated with higher levels of substance use, higher levels of community-violence witnessing, and lower levels of family support.

**Conclusions:**

The findings suggest the importance of considering gender-related differences in the effects of risk and protective factors when designing and implementing prevention programs. In interventions for both male and female adolescents, prevention of substance use and risky sexual behavior should be integrated. For boys, efforts should focus on the reduction of peer delinquency, while, for girls, improvement of family support should be emphasized.

## Background

Quantitative and qualitative studies of the sexual knowledge and practices of adolescents reveal that a substantial number of boys and girls in many developing countries engage in sexual intercourse before their 15^th ^birthdays [1]. Early and unprotected sexual initiation can trigger a succession of harmful physical, emotional, and social outcomes, especially for girls [2]. Moreover, compared with adults, adolescents are less likely to have the foresight, skills, cognitive maturity, information, and support they need to protect themselves from unwanted pregnancy, HIV, and sexually transmitted infections [3]. In addition, the rising number of new HIV infections among this young demographic signals an urgent need to identify behavior and situations that contribute to sexual and reproductive health in adolescence [1].

In Cambodia, progress has been made in the battle against HIV. The estimated prevalence of HIV among adults aged 15 to 49 decreased from 2.0% in 2001 to 0.9% in 2007 [4]. However, new challenges and complexities surrounding HIV transmission are emerging. HIV transmission is increasing between men and their regular partners [5]. Whereas marriage traditionally marks the onset of sexual activity for the majority of females, premarital sexual activity is common among males [6]. Furthermore, visits to commercial sex workers among young men is widespread and socially accepted [6]. These young men may subsequently have sex with other non-commercial partners, such as their female schoolmates, and thus become a bridge population for HIV transmission. What is also clear from existing studies is that Cambodian adolescents have been vulnerable to disturbing trends of substance use and the consequent health outcomes including risky sexual behavior [7]. Yet relatively little is known about the sexual behavior and reproductive health needs of adolescents in Cambodia.

Previous studies have identified risk and protective factors in different domains that predict risky sexual behavior in adolescents. The indentified risk factors include substance use [8], peer delinquency [9,10], depression [11], and exposure to community violence [12]. Regarding protective factors, risky sexual behavior has been negatively associated with parental monitoring [13], parental trust [14], family meal frequency [15], family structure [16], and school engagement [9]. Thus the etiology of adolescent risky sexual behavior is multi-factorial. Yet there has been debate over which domains of risk or protection are more closely related to risky sexual behavior among male and female adolescents compared with others [17].

Including all possible risk and protective factors in a single study would present a formidable task; therefore, many scholars recommend that theory-driven models be used to direct the study of adolescent health risk behavior [18]. In this study, we used the Social Development Model (SDM), which incorporates both risk and protective factors into a general theory of adolescent risky behavior [19]. The SDM proposes that adolescents learn patterns of behavior from socializing agents in four main contexts: parents, peers, schools, and community. The SDM has been tested and supported in a variety of adolescent outcomes [20,21] and across different gender as well as socio-demographic groups [22,23]. The objective of this study was to identify the operation of risk and protective factors in individual, family, peer, school, and community domains in predicting risky sexual behavior among male and female adolescent students in Cambodia.

## Methods

### Study site and sampling procedure

From October 2007 to January 2008, we conducted this study in Battambang provincial town located in the north-west part of Cambodia. "Probability Proportional to Size" sampling method was used to select 10% of male and female adolescents from a list of students in each classroom from grades 7 through 12 for each of the 11 schools. Out of 2,096 students selected, 118 (5.7%) were absent on the day of data collection, 23 (1.1%) were not permitted to participate by their parents or guardians, and 12 (0.6%) were excluded because more than one-third of the questionnaire was not completed. In our analysis, we included only students in grades 10 through 12 (n = 1,049) due to the low prevalence of risky sexual behavior among students in grades 7 through 9 (n = 894).

### Study procedure

This study was approved by the Ethical Committee of the University of Tokyo, Japan and the National Ethics Committee for Health Research, Ministry of Health, Cambodia. We initially developed the survey questionnaire in English and then translated it into Khmer, the national language of Cambodia. Another translator back-translated the questionnaire to ensure that the "content and spirit" of every original item was maintained. Some necessary modifications were made based upon comments from public health and education professionals in Cambodia. Prior to the main data collection phase, we conducted a pilot study among 273 students before constructing the final questionnaire.

One week in advance of the day designated for data collection, school principals sent a letter to the parents or guardians of the selected students. In the letter, we explained the study and gave the parents or guardians the opportunity to opt out on behalf of their children. Students also had an opportunity to refuse or to discontinue participation at any time. We ensured confidentiality by removing all personal identifiers from the questionnaires. At each school, the questionnaire was administered in a common hall. The principal investigator and two research assistants informed the participants carefully about the study and were available throughout the administration of the questionnaires to answer questions from individual students.

### Variables and measurements

The expected risk factors included substance use, depression, peer delinquency, family-violence victimization, family-violence witnessing, community-violence victimization, and community-violence witnessing. Protective factors included family support function, frequency of family dinner, and school attachment. For the details of questionnaire used in this study please see 'Additional file 1.'

#### Demographic characteristics

We collected information on age, gender, school grade, family structure (living with two-parents, a single-parent, or other adult guardians), parental occupation, parental education, monthly family income, and family accommodation (own house, rented house, relative's house, or public places). Parental education was categorized into [1 = 9 years or less, 2 = 10 years more, and 3 = don't know] and monthly family income into [1 = ≤ US$ 100, 2 = US$ 101- 300, and 3 = > US$ 300].

#### Risky sexual behavior

Four items constituting a risky sexual behavior scale were adapted from a previous study [10]. We asked whether the participants had engaged in sexual intercourse during the past three months, the number of sex partners during the past three months, the age at first experience of sexual intercourse, and if the participants used a condom in their last instance of sexual intercourse. Regarding age at first experience of sexual intercourse, the responses were coded as follows: 0 if the participants never had sex, 1 if the age reported was 17 or older, 2 if the age reported was 16, 3 if the age reported was 15, 4 if the age reported was 14, and 5 if the age reported was 13 or younger. Regarding number of sex partners, the responses were coded 0 if the participants never had sex, 1 if the number reported was 1, and 2 if the number reported was 2 or more. Regarding condom use, the responses were coded 0 if the participants never had sex, 1 if the answer was "yes," and 2 if the answer was "no." The total score of these four measures was then calculated, with higher scores indicating higher levels of risky sexual behavior (Cronbach's α in our study was 0.90).

#### Substance use

Regarding substance use, we modified related parts of the 2007 Youth Risk Behavior Survey Form, from the Center for Disease Control Youth Risk Behavior Surveillance System [24]. We collected information regarding the use of illicit drugs (methamphetamine, heroin, ecstasy, inhalants, cocaine, or marijuana), alcohol drinking (at least a full glass of beer, wine, or liquor), and smoking (at least a whole cigarette) during the past three months. All response options were dichotomous (0 = no, 1 = yes). The total score of the three measures was calculated, with higher scores indicating higher levels of substance use (Cronbach's α in our study was = 0.72).

#### Depression

To measure depression, we used the Asian Adolescent Depression Scale (AADS) [25]. The scale comprises four dimensions: negative self-evaluation (seven items), negative affect (five items), cognitive inefficiency (four items), and lack of motivation (four items) with a five-point response option ranging from (1) "Strongly disagree" to (5) "Strongly agree." The total AADS score is the sum of the 20 items, with a range from 20 to 100. A higher score indicates a higher level of depression, and an adolescent with a total score of 80 or above would be diagnosed as depressed (Cronbach's α in our study was 0.88).

#### Peer delinquency

Peer delinquency was assessed using a scale adapted from a previous study [10]. Participants were asked how many of their friends engaged in various delinquent activities in the previous six months such as cutting school, damaging property, stealing, joyriding, hitting, attacking, using weapons, using drugs, or having sex with someone. The response categories included: "0 = none," "1 = few," "2 = half," "3 = most," and "4 = all." A higher score indicated a higher level of peer delinquency (Cronbach's α in our study was 0.91).

#### Family violence

Regarding students' victimization by and witnessing of family violence, we used two yes/no questions adapted from a previous study [26]: (1) "During the previous two years, has there been any time when you were hit, slapped, or received any physical punishment from a parent or other adult guardian?" and (2) "During the previous two years, have you seen or heard one of your parents or guardians being hit, slapped, or otherwise physically hurt by another adult in your family?"

#### Community violence

We adapted six victimization and six witnessing items from previous research to collect information about exposure to community violence [27]. Each item had a binary (0 = no/1 = yes) response format and addressed exposure during the previous two years. Victimization items (Cronbach's α in our study was 0.70) were: have you been (1) "beaten up or mugged;" (2) "threatened with serious physical harm;" (3) "shot or shot at with a gun;" (4) "attacked or stabbed with a knife;" (5) "chased by gangs or individuals;" and (6) "seriously wounded in an act of violence." For community-violence witnessing, we also asked students whether they had witnessed the same seven types of violence during the past two years (Cronbach's α in our study was 0.75).

#### Family support function

We adapted 17 items from the Family Support Function Scale (FSFS) to measure family support [28]. The scale combines three dimensions including positive functioning, common responsibility, and negative functioning using four response options: "Rarely or never," "Sometimes," "Often," and "Almost always." The total FSFS score was calculated as the sum of the 17 items. A higher score indicated a more supportive family (Cronbach's α in our study was 0.84).

#### Family dinner frequency

To assess family dinner frequency, we asked "In an average week, how many times do all of the people in your family who live with you eat dinner together?" [15]. The response options were: [0 = 0- 1 time per week, 1 = 2- 4 times per week, and 2 = 5- 7 times per week].

#### School attachment

We measured school attachment using seven items adapted from a previous study [29]. The seven items- "I like school," "My teachers like me," "I like my teachers," "School is fun," "I am accepted in school," "I feel like an outsider in school," and "I feel like I fit in at school"- were measured on a 4-point scale that included "Not at all," "Not much," "Some," and "A lot" as response choices. The item- "I feel like an outsider in school" was reverse coded. A higher score indicates stronger school attachment (Cronbach's α in our study was 0.65).

### Data analysis

We first calculated the total scores on all scales used in this study. To assess bivariate associations between demographic characteristics and risky sexual behavior, Chi-square test or Fisher's exact test were used for categorical variables, and *t-*test or one-way analysis of variance (ANOVA) was used for continuous variables. Bivariate regression analyses were conducted to assess the unadjusted associations between risk and protective factors and risky sexual behavior stratified by gender. Multiple linear regression analyses were then performed separately for males and females to detect the independent associations between all expected predictors and risky sexual behavior. Risk and protective factors and demographic characteristics were entered simultaneously in the models if they were found to have associations with risky sexual behavior at a level of *p *≤ .2 in bivariate analyses. No multicollinearity was detected in the model. We used SPSS version 15.0 (SPSS Inc, Chicago, IL) for all statistical analyses.

## Results

### Descriptive statistics

Of the 1,049 students surveyed, 56.5% were male, and the mean age was 17.6 years (SD = 1.3). Paternal occupations included farmer (43.4%), self-owned business (24.9%), government officers (21.4%), taxi drivers (2.8%), and retired (7.5%). Participants lived with two parents (70.1%), a single-parent (15.8%), or other adult guardians (step-parent, relatives, or in an orphanage) (14.1%). Eighty-eight percent of participants lived in their own family home, and 54.4% reported that their monthly family income was US$ 100 or less. Regarding parental education, 49.4% of fathers and 45.5% of mothers had completed ten years or more of formal education. Regarding sexual experiences, 12.7% of the participants reported sexual intercourse during the past three months. Out of these sexually active students, 34.6% had two or more sex partners during the past three months, and 52.6% did not use a condom during their last instance of sexual intercourse.

### Bivariate analyses

Table 1 shows that boys were significantly more likely than girls to have had sexual intercourse (OR = 3.9, 95% CI = 2.47-6.12) and two or more sex partners (OR = 4.6, 95% CI = 2.02-10.28) during the past three months. However, girls were significantly more likely than boys to have reported that they or their partners did not use a condom during their last sexual intercourse (OR = 3.7, 95% CI = 1.30-10.78). In terms of age distribution, students in the age group of 17 and older were more likely to have had sexual intercourse (OR = 1.5, 95% CI = 1.02-2.27) and to have had two or more sex partners during the past three months (OR = 3.1, 95% CI = 1.68-5.62) compared to those aged 16 or younger. As shown in Table 2, the mean score on the risky sexual behavior scale was significantly higher among students in the age group of 17 or older (*p *< 0.001), among boys (*p *< 0.001), and among students whose fathers were farmers (*p *< 0.038) relative to their comparison groups.

**Table 1:** Prevalence of risky sexual behavior stratified by gender

	Gender (n, %)		
	**Male**	**Female**	**OR (95% CI)**

Sexual intercourse- past 3 months	108 (18.4)	25 (5.5)	3.9 (2.47-6.12)

Two or more sex partners *	39 (36.1)	7 (28.0)	4.6 (2.02-10.28)

No condom use- last sex *	33 (36.7)	13 (68.4)	3.7 (1.30-10.78)

n = Number; OR = Odds ratio; CI = Confidence interval.

* Data were only from students who had sexual intercourse during the past 3 months.

**Table 2:** Comparisons of mean scores of risky sexual behavior by socio-demographic characteristics

		Risky sexual behavior		
	**n (%)**	**Mean**	**SD**	**p-value**

Age				

≤ 17 years	802 (76.5)	2.3	1.1	< 0.001

≥ 18 years	246 (23.5)	2.7	1.6	

Gender				

Male	590 (56.5)	2.6	1.5	< 0.001

Female	455 (43.5)	2.1	0.8	

Family structure				

Two-parents	733 (70.1)	2.4	1.2	0.547

Other *	313 (29.9)	2.5	1.3	

Father's occupation				

Farmer	399 (38.2)	2.5	1.4	0.038

Other^†^	645 (61.8)	2.4	1.2	

Father's education				

≤ Grade 9	287 (27.5)	2.4	1.3	0.367

≥ Grade 10	280 (26.7)	2.5	1.3	

Don't know	481 (45.8)	2.4	1.2	

Mother's education				

≤ Grade 9	403 (38.5)	2.4	1.3	0.280

≥ Grade 10	176 (16.8)	2.5	1.4	

Don't know	468 (44.7)	2.4	1.2	

Monthly family income				

≤ US$ 100	561 (54.4)	2.3	1.1	0.081

> US$ 100	470 (45.6)	2.5	1.3	

Type of family home				

Own home	924 (88.3)	2.4	1.2	0.138

No family home	122 (11.7)	2.6	1.4	

n = Number; SD = Standard deviation.

* "Other" included living with a single parent, with relatives, or in an orphanage.

^† "^Other" included being self-employed, an office worker, a government officer, or a taxi driver.

Table 3 shows the effects of risk and protective factor on risky sexual behavior before adjustment. Among boys, higher likelihood of risky sexual behavior was significantly associated with risk factors including higher level of substance use (*β *= 0.222, SE = 0.037, *p *< 0.001) and higher level of peer delinquency (*β *= 0.195, SE = 0.008, *p *< 0.001). Among girls, higher likelihood of risky sexual behavior was significantly associated with higher level of substance use (*β *= 0.191, SE = 0.127, *p *< 0.001), higher level of depression (*β *= 0.160, SE = 0.003, *p *< 0.001), higher level of peer delinquency (*β *= 0.114, SE = 0.005, *p *= 0.016), higher level of community-violence victimization (*β *= 0.173, SE = 0.047, *p *< 0.001), higher level of community-violence witnessing (*β *= 0.166, SE = 0.023, *p *< 0.001), lower level of family support function (*β *= - 0.217, SE = 0.004, *p *< 0.001), lower level of school attachment (*β *= - 0.179, SE = 0.014, *p *< 0.001), and more frequent sport practice (*β *= 0.096, SE = 0.073, *p *= 0.044).

**Table 3:** Results of bivariate regression analyses showing risk and protective factors associated with risky sexual behavior, stratified by gender

*Expected risk and protective factors *	*β*	SE	*p- *value
**Male**			

Substance use	0.222	0.037	< 0.001

Depression	0.079	0.005	0.063

Peer delinquency	0.195	0.008	< 0.001

Family violence- victimization	0.040	0.132	0.345

Family violence- witnessing	0.008	0.126	0.840

Community violence- victimization	0.066	0.062	0.119

Community violence- witnessing	0.073	0.038	0.083

Family support function	- 0.073	0.008	0.082

School attachment	- 0.029	0.022	0.485

Family dinner frequency	0.036	0.079	0.391

**Female**			

Substance use	0.191	0.027	< 0.001

Depression	0.160	0.003	0.001

Peer delinquency	0.114	0.005	0.016

Family violence- victimization	0.054	0.090	0.259

Family violence- witnessing	0.102	0.074	0.032

Community violence- victimization	0.173	0.047	< 0.001

Community violence- witnessing	0.166	0.023	< 0.001

Family support function	0.217	0.004	< 0.001

School attachment	- 0.179	0.014	< 0.001

Family dinner frequency	- 0.061	0.045	0.198

SE = Standard error.

### Multivariate analyses

The results of multiple linear regression analyses are shown in Table 4. Among boys, after controlling for other covariates, higher likelihood of risky sexual behavior remained significantly associated with higher levels of substance use (*β *= 0.193, SE = 0.040, *p *< 0.001), higher levels of peer delinquency (*β *= 0.109, SE = 0.009, *p *= 0.023), and higher family income (*β *= 0.130, SE = 0.130, *p *= 0.004). Among girls, higher likelihood of risky sexual behavior remained significantly associated with higher levels of substance use (*β *= 0.174, SE = 0.024, *p *< 0.001), higher levels of community-violence witnessing (*β *= 0.136, SE = 0.023, *p *= 0.010), and lower levels of family support (*β *= - 0.197, SE = 0.004, *p *< 0.001). These results could be interpreted that, for example, for each point increase in the substance use scale, the risky sexual behavior increases by 0.193 units among boys and by 0.174 units among girls.

**Table 4:** Results of multiple linear regression analyses showing independent predictors of risky sexual behavior, stratified by gender

*Expected risk and protective factors*	β	SE	*p- *value
**Male (n = 493)**			

Substance use	0.193	0.040	< 0.001

Depression	0.010	0.005	0.831

Peer delinquency	0.109	0.009	0.023

Community-violence victimization	0.053	0.064	0.250

Community-violence witnessing	0.012	0.042	0.802

Family support function	- 0.039	0.008	0.377

Higher family income	0.130	0.130	0.004

Father's occupation as a farmer - 0.087	0.133	0.055	

Age	0.081	0.047	0.080

**Female (n = 414)**			

Substance use	0.174	0.024	< 0.001

Depression	0.026	0.003	0.603

Peer delinquency	- 0.041	0.005	0.424

Family-violence -witnessing	0.013	0.069	0.796

Community-violence victimization	0.038	0.045	0.445

Community-violence witnessing	0.136	0.023	0.010

Family support function	0.197	0.004	< 0.001

School attachment	- 0.056	0.014	0.271

More frequent family dinner	0.062	0.027	0.073

Father's occupation as farmer	0.008	0.065	0.862

Higher family income	0.019	0.067	0.703

Age	0.028	0.026	0.559

SE = Standard error.

## Discussion

This study provides insight into the operation of risk and protective factors in different domains to predict risky sexual behavior among adolescents in Cambodia. A unique aspect of this study is that we were able to examine several predictors of risky sexual behavior among male and female students simultaneously controlled for the effects of other covariates in multivariable regression models. By using this method, we were able to uncover different characteristics of the associations across genders. Among boys, significant predictors of risky sexual behavior included substance use and peer delinquency. None of the protective factors remained significantly associated with risky sexual behavior among boys. Among girls, significant associations were found with risk factors, including substance use and community-violence witnessing, and protective factors including family support function.

Substance use was one of the most powerful predictors of risky sexual behavior among both boys and girls in this study. The finding extends the widespread evidence that substance use and risky sexual practices tend to co-occur among adolescents [30]. This is also consistent with findings from previous research in developing countries, which has linked illicit drug or alcohol use with adolescent premarital sex and non-use of condoms [31]. The most frequently cited explanation for the link between substance use and risky sexual behavior is sensation-seeking behavior, which is defined as a disposition characterized by the tendency to pursue novel, exciting, and optimal levels of stimulation [32]. Another possible explanation for this co-variation is that intoxication with substances, such as alcohol or methamphetamine, may have a disorganizing effect on cognitive functions leading to poor decision-making vis-à-vis involvement with risky sexual behavior [33]

In this study, peer delinquency retained its significant association with risky sexual behavior among boys after controlling for the effects of other covariates. These findings support the existing research studies which suggest that the correlations between peer relationships and adolescent health risky behavior become salient due to the increases in peer affiliations that typically characterize adolescence [34]. Peer influence seems to be the most important factor in adolescents' decision-making and risk-taking behavior [35]. Affiliation with delinquent peers and having sexually active friends were two significant factors for initiation of early sexual intercourse among adolescents [31,34].

The structure of peer pressure has been used to explain these associations. Peer pressure is defined as pressure from peers to "do something or to keep from doing something else, no matter if you personally want to or not" [36]. In this way, peer delinquency can act as a source of pressure for adolescents to become involved in risky sexual practices. Being friends with delinquent peers predicts risky behavior beyond contributions from other factors [37]. Delinquent peers provide adolescents the opportunity to expose themselves to health risks by contributing to poor decision-making [37]. Delinquent peers are also more likely to promote maladaptive practices and adolescents who affiliate with such peers may be influenced or pressured into behaving similarly [10].

Regarding exposure to violence, risky sexual behavior remained significantly associated with community-violence witnessing among girls. These findings are supported by stress and coping theory which posits that violence exposure precipitates maladaptive coping strategies that serve to elude thoughts and feelings about the stressors [38]. Consistent with this notion, our findings may indicate that adolescent girls who had been exposed to violence practice risky behavior, such as early sexual initiation or unprotected sexual intercourse, to self-medicate the distress caused by their experience of neighborhood violence.

It is worth noting that the protective factors examined in this study had only limited protective effects against risky sexual behavior. Family support function remained significantly associated with risky sexual behavior only among girls, while other protective factors did not remain significant predictors in both genders. The non-significant associations do not support a growing body of literature which suggests that protective factors are also important in adolescent health outcomes, either by exerting positive influences with a canceling effect of risk factors, or by buffering the negative effects of risk factors [39].

The non-significant findings in this study can be explained by assuming that the effects of protective factors on adolescent behavior were channeled by other factors such as deviant peer involvement owing to the decreases in family bonding and increases in peer affiliation during adolescence [10]. The adolescents' closeness to their parents and teachers plays a less central role in their lives as they progress through adolescence into young adulthood [40,41]. The inconsistency of the findings might be caused in part by the differences in the characteristics of the participants and our inability to control for potential third factors.

Nevertheless, our findings do not necessarily imply that family and school influences are not important. Parents and teachers may be important in influencing adolescents' early orientation toward peers [42]. Adolescents who have harmonious relationships with their family members are less likely to associate with delinquent peers [43]. Parents also provide social norms related to appropriate behavior, as well as having an important function in the supervision and monitoring of adolescents' tendencies towards inappropriate behavior. This relationship is more complex, as weakened bonds to family and school and risky behavior operate in an interactional manner, with mutual and simultaneous influences on one another [44]. Furthermore, the magnitude and significance of covariates generally depend on the choice and availability of variables. Interpretation of the relative influence of protective factors should thus be approached with great caution.

In this study, several limitations merit discussion. First, the study's cross-sectional design prohibited us from identifying causal relationships between the predictors and risky sexual behavior [37]. For example, it remained unclear whether negative life events, such as substance use, contributed to risky sexual involvement, or, conversely, whether risky sexual practices were the cause of the substance use. A study with a longitudinal design is needed to address this shortcoming. The second limitation concerns the representativeness of the sample. Although students from a wide range of socio-economic background were included, the findings may not necessarily generalize to students living in more rural areas or out-of-school adolescents whose living conditions and life style may be different.

Third, as with any self-reported measures, there are inherent biases and the potential for both underreporting and over-reporting in the variables [10]. Given the cultural norms governing sexual behavior, it is likely that the gender difference is caused in part by over-reporting by boys and under-reporting by girls [6]. Fourth, findings from our study might be limited by the unknown reliability and validity of the scales which were adapted from previous studies conducted in developed countries. The final limitation concerns the fact that we collected only behavioral data which may not reflect the adolescents' actual risk for HIV or sexually transmitted infections.

## Conclusion

Despite these limitations, our findings contribute to the literature in several ways. The findings indicate that substance use and peer delinquency were the most important predictors of risky sexual behavior among male adolescents, while substance use, community-violence witnessing, and family support function play important roles in predicting risky sexual behavior among female adolescents. Our results suggest the importance of considering gender-related differences when designing and implementing prevention programs. Interventions that emphasize different domains of the risk and protective factors in an integrated manner across genders may be the most effective. Among both boys and girls, reduction of substance use and risky sexual behavior should be incorporated into preventive programs. At the same time, reduction of peer delinquency should be the primary focus for interventions targeting boys, while improvement of family support should be emphasized in interventions targeting girls.

## Competing interests

The authors declare that they have no competing interests.

## Authors' contributions

SY conceived the research questions, designed the study, conducted preparatory field works, collected data, analyzed data, and drafted the manuscript for publication. KCP was involved in revisions of the research proposal, data analysis, and preparation of the manuscript for publication. JY was involved in revisions of the research proposal, data analysis, and revisions of the manuscript for publication. PHP was involved in data analysis and revisions of the manuscript for publication. SY was involved in preparatory field works, data collection, and revisions of the manuscript for publication. MJ was involved in revisions of the research proposal, data analysis, and revisions of the manuscript for publication. All authors read and approved the final manuscript.

## Pre-publication history

The pre-publication history for this paper can be accessed here:

http://www.biomedcentral.com/1471-2458/10/477/prepub

## Supplementary Material

Additional file 1 **Survey questionnaire**. Details of the questionnaire used in this study Click here for file
