# Supplementary material for: Role of risk and protective factors in risky sexual behavior among high school students in Cambodia
Source: BMC Public Health. 2010 Aug 12;10:477. doi: 10.1186/1471-2458-10-477 (PMC2928204; doi:10.1186/1471-2458-10-477)
Supplement: Additional file 1 — Survey questionnaire. Details of the questionnaire used in this study [file 1471-2458-10-477-S1.DOC]

***Additional File 1: Questionnaire***

**I. About demographic background**

1. How old are you? ……………………. Years

2. What is your sex? A. □ Male B. □ Female

3. What is your father’s job? A. □ Died B. □ Farmer C. □ self-owned business

D. □ Govnt officer E. □ Taxi driver F. □ Retired professional G. □ Other (Specify………………………………………)

4. What is your mother’s job? A. □ Died B. □ Farmer

C. □ self-owned business D. □ Government officer E. □ Housewife F. □ Retired professional G. □ Other (Specify……...................................)

5. Do you live with….?

A. □ Single-parent B. □ Two parents C. □ Step parent

D. □ Relatives E. □ Other (Specify…………)

6. How many years of schooling had your father completed?

A. □ No formal education B. □ 1- 5 years C. 6- 9 years

C. □ 10- 12 years D. □ More than 12 years

7. How many years of schooling had your mother completed?

A. □ No formal education B. □ 1- 5 years C. 6- 9 years

C. □ 10- 12 years D. □ More than 12 years

8. What type of accommodation is your family living in?

A. □ Own house B. □ Rented house C. □ Someone else’s house

D. □ Shelter/group house E. □ Street F. □ Other (Specify………)

9. How much is the estimated monthly income of your family?

A. □ Less than US$100 B. □ US$ 100- 200 C. □ US$201- 300

D. □ US$ 300- 400 E. □ US$ 400-500 F. □ More than US$ 500

10. During the past one year, in an average week, how many times do all of the people in your family who live with you eat dinner together?

A. □ 0– 1 time B. □ 2- 4 times C. □ 5- 7 times

**II. Depression**

During the preceding two weeks…

1. I felt that I am not as good as others. A. □ Strongly disagree B. □ Disagree

C. □ Neither agree nor disagree. D. □ Agree E. □ Strongly agree

2. I felt that I am not wanted. A. □ Strongly disagree B. □ Disagree

C. □ Neither agree nor disagree. D. □ Agree E. □ Strongly agree

3. I felt hopeless. A. □ Strongly disagree B. □ Disagree

C. □Neither agree nor disagree D. □ Agree E. □ Strongly agree

4. Nothing works out right for me. A. □ Strongly disagree B. □ Disagree

C. □ Neither agree nor disagree. D. □Agree E. □ Strongly agree

5. I was confused about what kind of person I am. A. □ Strongly disagree B. □ Disagree C. □ Neither agree nor disagree. D. □ Agree E. □ Strongly agree

6. I did not get satisfaction from what I do. A. □ Strongly disagree B. □ Disagree

C. □ Neither agree nor disagree D. □ Agree E. □ Strongly agree

7. I felt that I have no control over what happens . A. □ Strongly disagree B. □ Disagree C. □ Neither agree nor disagree D. □ Agree E. □ Strongly agree

8. I had thought about dying. A. □ Strongly disagree B. □ Disagree

C. □ Neither agree nor disagree D. □ Agree E. □ Strongly agree

9. I often felt like crying . A. □ Strongly disagree B. □ Disagree

C. □ Neither agree nor disagree D. □ Agree E. □ Strongly agree

10. I felt sad most of the time. A. □ Strongly disagree B. □ Disagree

C. □ Neither agree nor disagree D. □ Agree E. □ Strongly agree

11. My heart felt heavy. A. □ Strongly disagree B. □ Disagree

C. □Neither agree nor disagree D. □ Agree E. □ Strongly agree

12. I was more badly tempered than before. A. □ Strongly disagree B. □ Disagree

C. □ Neither agree nor disagree D. □ Agree E. □ Strongly agree

13. I took a long time to decide on things. A. □ Strongly disagree B. □ Disagree

C. □ Neither agree nor disagree D. □ Agree E. □ Strongly agree

14. I took long time to get things done. A. □ Strongly disagree B. □ Disagree

C. □Neither agree nor disagree D. □ Agree E. □ Strongly agree

15. I could not think well. A. □ Strongly disagree B. □ Disagree

C. □ Neither agree nor disagree D. □ Agree E. □ Strongly agree

16. I could not concentrate on my studies as much as I used to. A. □ Strongly disagree B. □ Disagree C. □ Neither agree nor disagree D. □ Agree E. □ Strongly agree

17. I felt that I have no energy to do things most of the time. A. □ Strongly disagree B. □ Disagree C. □ Neither agree nor disagree D. □ Agree E. □ Strongly agree. 18. I felt tired most of the time. A. □ Strongly disagree B. □ Disagree

C. □ Neither agree nor disagree D. □ Agree E. □ Strongly agree

19. I did not feel like doing anything. A. □ Strongly disagree B. □ Disagree

C. □Neither agree nor disagree D. □ Agree E. □ Strongly agree

20. I did not like going out with friends or meeting people. A. □ Strongly disagree

B. □ Disagree C. □ Neither agree nor disagree D. □ Agree E. □ Strongly agree

**III. Peer delinquency**

During the past 6 months, how many of your friends engaged in the following delinquent activities?

1. Skipped a day of school without a real excuse? A. □ None B. □ Few

C. □ Half D. □ Most E. □ All

2. Purposely damaged or messed up public properties? A. □ None B. □ Few

C. □ Half D. □ Most E. □ All

3. Took something of value which did not belong to them? A. □ None B. □ Few

C. □ Half D. □ Most E. □ All

4. Rode a motorbike or drove a car fast for joy? A. □ None B. □ Few

C. □ Half D. □ Most E. □ All

5. Beat up someone on purpose? A. □ None B. □ Few

C. □ Half D. □ Most E. □ All

6. Got involved in a physical fight with a gang or group of friends? A. □ None B. □ Few

C. □ Half D. □ Most E. □ All

7. Carried a weapon such as a gun, knife, or club? A. □ None B. □ Few

C. □ Half D. □ Most E. □ All

8. Drank five or more cans of beer, or glasses of wine? A. □ None B. □ Few

C. □ Half D. □ Most E. □ All

9. Used drugs such as Yama/Yaba, marijuana, injected drugs, sniffed glue? A. □ None

B. □ Few C. □ Half D. □ Most E. □ All

10. Used dirty language or swear words? A. □ None B. □ Few

C. □ Half D. □ Most E. □ All

11. Argued or fought with their mother? A. □ None B. □ Few

C. □ Half D. □ Most E. □ All

12. Argued or fought with their father? A. □ None B. □ Few

C. □ Half D. □ Most E. □ All

13. Ran away from home? A. □ None B. □ Few

C. □ Half D. □ Most E. □ All

14. Stayed out later than their parents said they should? A. □ None B. □ Few

C. □ Half D. □ Most E. □ All

15. Had sexual relation with someone? A. □ None B. □ Few

C. □ Half D. □ Most E. □ All

16. Tried to get something by lying a person about what they would do for him or who they were? A. □ None B. □ Few C. □ Half D. □ Most E. □ All

17. Were late in submitting homework? A. □ None B. □ Few

C. □ Half D. □ Most E. □ All

**IV. Exposure to family violence**

During the previous two years,

1. Has there been any time when you were hit, slapped, kicked or received any physical punishment from a parent or other adult guardian? A. □ No B. □ Yes

2. Have you ever seen or heard one of your parents or guardian being hit, slapped, kicked, or otherwise physically hurt by another adult in your family? A.□ No B.□ Yes

**V. Exposure to Community violence**

***Community-violence victimization***

During the previous two years, have you been:

1. Beaten up or mugged? A. □ No B. □ Yes

2. Threatened with serious physical harm? A. □ No B. □ Yes

3. Shot or shot at with a gun? A. □ No B. □ Yes

4. Attacked or stabbed with a knife? A. □ No B. □ Yes

5. Chased by gangs or individuals? A. □ No B. □ Yes

6. Seriously wounded in an accident of violence? A. □ No B. □ Yes

***Community-violence witnessing***

During the previous two years, have you witnessed somebody being:

1. Beaten up or mugged? A. □ No B. □ Yes

2. Threatened with serious physical harm? A. □ No B. □ Yes

3. Shot or shot at with a gun? A. □ No B. □ Yes

4. Attacked or stabbed with a knife? A. □ No B. □ Yes

5. Chased by gangs or individuals? A. □ No B. □ Yes

6. Seriously wounded in an accident of violence? A. □ No B. □ Yes

**VI. Family support function**

During the past one year…

1. Family members really help each other when in trouble.

A. □ Rarely or never B. □ Sometimes C. □ Often D. □ Almost always

2. Family members actively discuss their problems and opinions with the others

A. □ Rarely or never B. □ Sometimes C. □ Often D. □ Almost always

3. Family members share interests and hobbies with each other.

A. □ Rarely or never B. □ Sometimes C. □ Often D. □ Almost always

4. Family members often spend free time with each other.

A. □ Rarely or never B. □ Sometimes C. □ Often D. □ Almost always

5. Our family usually keeps a harmonious atmosphere.

A. □ Rarely or never B. □ Sometimes C. □ Often D. □ Almost always

6. Family members try to control themselves when a conflict appears.

A. □ Rarely or never B. □ Sometimes C. □ Often D. □ Almost always

7. Family members often pursue learning new knowledge.

A. □ Rarely or never B. □ Sometimes C. □ Often D. □ Almost always

8. When a family member meets trouble, family members would actively help him/her.

A. □ Rarely or never B. □ Sometimes C. □ Often D. □ Almost always

9. When a family member feels unhappy, another family member always knows why.

A. □ Rarely or never B. □ Sometimes C. □ Often D. □ Almost always

10. All family members speak openly.

A. □ Rarely or never B. □ Sometimes C. □ Often D. □ Almost always

11. Family members often talk to each other about school, life, and work

A. □ Rarely or never B. □ Sometimes C. □ Often D. □ Almost always

12. Family conflicts can be easily solved or controlled through communication.

A. □ Rarely or never B. □ Sometimes C. □ Often D. □ Almost always

13. Our family does things together.

A. □ Rarely or never B. □ Sometimes C. □ Often D. □ Almost always

14. Family members, including children, share household responsibilities.

A. □ Rarely or never B. □ Sometimes C. □ Often D. □ Almost always

15. We feel bored at home.

A. □ Rarely or never B. □ Sometimes C. □ Often D. □ Almost always

16. Family members like to discuss personal matters with friends other than family members.

A. □ Rarely or never B. □ Sometimes C. □ Often D. □ Almost always

17. Family members hide their opinions when conflict occurs.

A. □ Rarely or never B. □ Sometimes C. □ Often D. □ Almost always

**VII. About school attachment**

1. I like school. A. □ Not at all B. □ Not much C. □ Some D. □ A lot

2. My teachers like me. A. □ Not at all B. □ Not much C. □ Some D. □ A lot

3. I like my teachers. A. □ Not at all B. □ Not much C. □ Some D. □ A lot

4. School is fun . A. □ Not at all B. □ Not much C. □ Some D. □ A lot

5. I am accepted at school. A. □ Not at all B. □ Not much C. □ Some D. □ A lot

6. I feel like an outsider at school. A.□ Not at all B. □ Not much C. □ Some D. □ A lot

7. I feel like I fit in at school. A. □ Not at all B. □ Not much C. □ Some D. □ A lot

***VIII. About sexual experiences***

1. During the past three months, did you have sexual intercourse? A. □ No B. □ Yes

2. How old were you when you had sexual intercourse for the first time?

A. □ I have never had sexual intercourse B................ years old

3. The last time you had sexual intercourse, did you or your partner use a condom?

A. □ I have never had sexual intercourse B. □ No C. □ Yes

***IX. About illicit drug use***

1. During the past three months, did you use any kind of illicit drugs (methamphetamine, heroin, ecstasy, inhalants, cocaine, or marijuana)? A. □No B. □ Yes

2. During the past three months, what kind of illicit drugs did you use?

A. □ I have never used any illicit drug B. □ Yama or Yaba (Methamphetamine) C. □Injected drug D. □ Sniffed glue E. □ Others (Specify…………..)

3. How old were you when you tried any kind of illicit drugs for the first time?

A. □ I have never tried any drug B. ……… years old

***X. About alcohol use***

1. During the past three months, did you drink at least a full glass of any kind of alcohol?

A. □ No B. □ Yes

2. How old were you when you drank at least a full glass of alcohol for the first time?

A. □ I have never drunk alcohol B ………... years old

***XI. About tobacco use***

1. During the past three months, did you smoke at least a whole cigarette?

A. □ No B. □ Yes

2. How old were you when you smoked at least a whole cigarette for the first time?

A. □ I have never smoked B……….. years old

*Thank you for your kind cooperation.*
